# Fault, error, and failure

**DOI:** 10.1002/acm2.70106

**Published:** 2025-04-21

**Authors:** Mohammad Bakhtiari

**Affiliations:** ^1^ Department of Radiation Oncology WellSpan Radiation Oncology Chambersburg Pennsylvania USA

1

The safe and quality delivery of radiation therapy relies on clear communication across the community. In safety and quality literature, we frequently face terms such as fault, error, failure, incidents, and accidents. Evidently, there is some inconsistency in the taxonomy of these terms in radiation oncology. For example, TG 100, following quality standards, categorizes these under failure, with faults as the initiating state.^1^ Conversely, TG 314 defines fault as the end‐state,^2^ and report number 394^3^ uses the terms error and failure interchangeably. The complexity increases in radiation oncology, as a sociotechnical system involving machines and humans, impacting patient safety. Integration of Artificial Intelligence (AI) into radiation oncology highlights the need for standardized data inputs for training to maximize AI benefits. This introductory article attempts to create a sense of urgency for a coalition to standardize these terms. It does not establish a standard itself. Instead, it makes a preliminary introduction to the terms:^4^

1. **Fault**: A fault is a *latent* defect or system‐level imperfection that may remain dormant until specific conditions or events trigger it. It represents a deviation from the intended design or function of a system.
2. **Error**: An error is an *internal* deviation that occurs when a fault is activated. It is a manifestation of a fault within the system that leads to incorrect or unintended behavior but may not necessarily be observable externally.
3. **Failure**: A failure is an *externally* observable system functionality breakdown when the system no longer meets its intended specifications or performs its required functions. The end state resulting from an error impacts system performance or safety.

The sequence and terminology are crucial: cyclically, through causation, a fault **activates,** triggering an error. Error **propagates** as errors. When an error is observed in the external environment, it becomes a failure. This failure can subsequently **cause** a fault in the system it serves, and the cycle continues.

Understanding systems and their boundaries is essential because the definitions of fault, error, and failure can vary based on whether they are internal or external to a system. A system is an entity that interacts with other entities, including hardware, software, humans, and the physical world. It is composed of components, each of which can be another system, creating a recursive structure that stops when a component is considered atomic. The system boundary is crucial as it defines the common frontier between the system and its environment, determining the inputs and outputs of the system. A fault can occur within this boundary, which is the cause of an error. This error propagates through the system's internal states and may eventually become visible as a failure in the system's external state. This failure, perceived at the service interface, can act as an external fault for another system, initiating a new cycle of fault, error, and failure. The line between error detection and error observation is subtle but significant. While error detection refers to identifying discrepancies internally within a system, error observation occurs when these discrepancies manifest externally, leading to an observable failure state.

From a safety perspective, if humans are involved in the system, failures can be categorized as follows: If a failure does not affect the human, it is considered a near miss. If failure affects humans but causes no harm, it is classified as an incident. If failure results in harm, it is deemed an accident.^5^, ^6^

Figure 1 provides a summary of these definitions. It illustrates the relationship between fault, error, and failure. The graph suggests that functional safety applies primarily to engineering or technical domains when no patient is involved. If a patient is engaged, functional safety falls under the broader category of patient safety.

**FIGURE 1:**
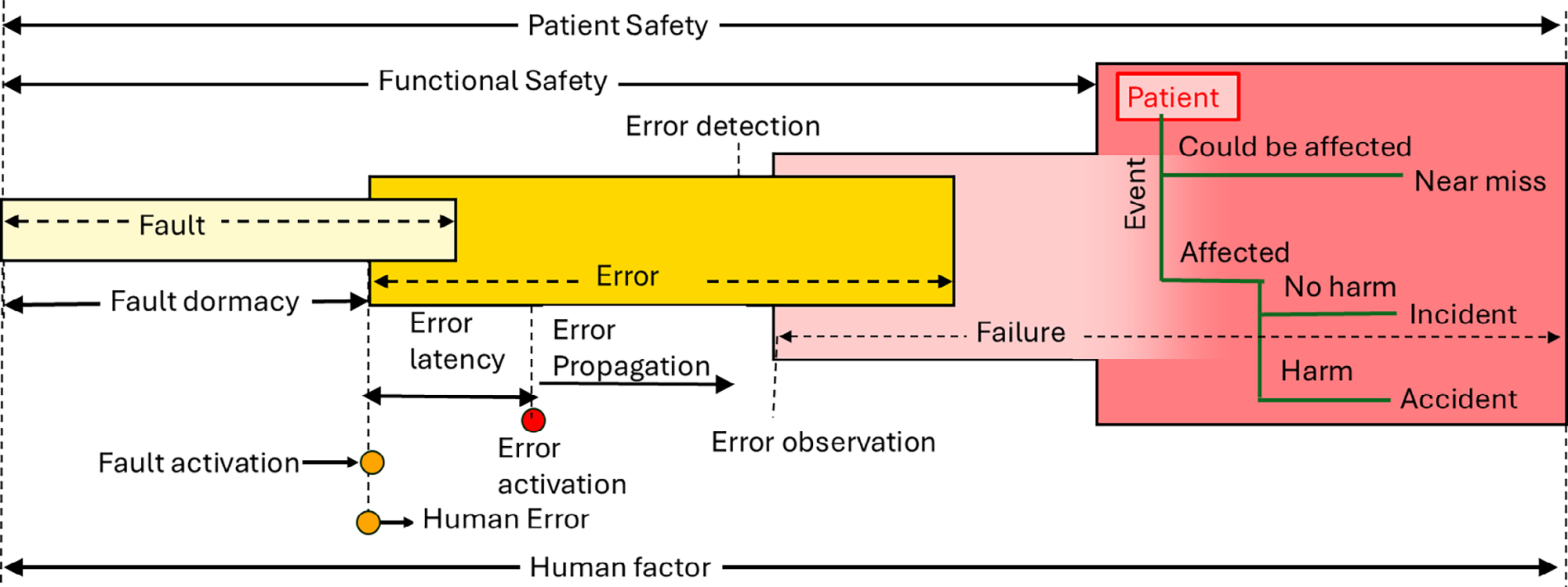
Definition of terms.

The human factor covers the entire system and timeline. Human errors happen in just a short amount of time or an instance.^7^

Figure 2 illustrates an example of a wrong couch density override to demonstrate that the fault creates an error, which propagates to the patient. If the error is observed, it becomes a failure and, depending on the beam intensity is categorized as a near miss, incident, or accident (adverse event). Detailed scenarios of the case are summarized in Table 1.

**FIGURE 2:**
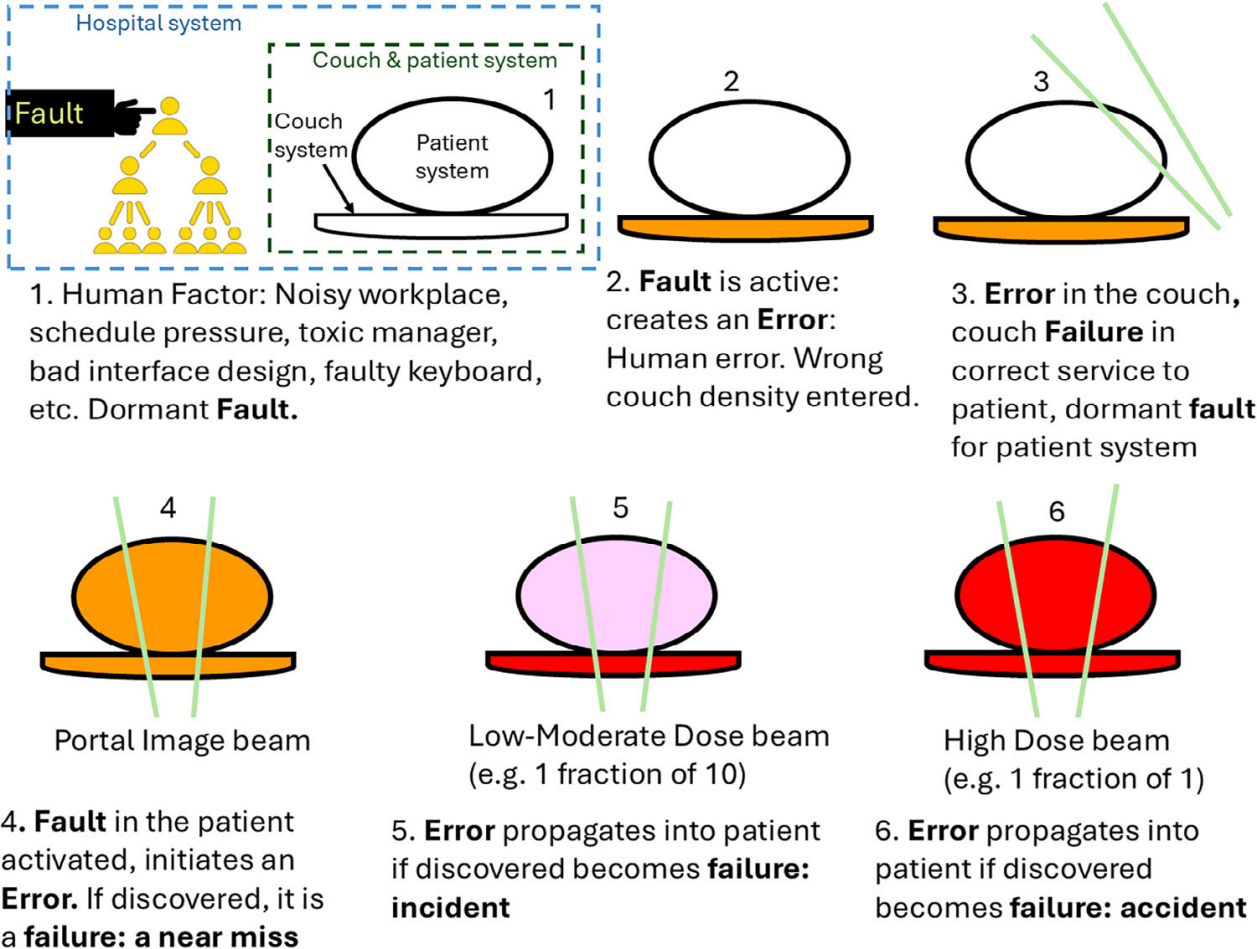
An example to illustrate the terms.

**TABLE 1:** The implications for cases in Figure 2.

Observation (cases 4–6)	Implication
Not found	If the couch and patient are considered one system, a density error in the couch propagates as a dose error to the patient. If systems are considered separate, the patient system perceives the error as a failure at the boundary. That becomes an external fault (wrong density) for the patient system which activates a dose error
Found before treatment	Failure in the couch system boundary is an external fault for the patient system, which initiates an error in the patient. It is observed: it is a failure. Patient involved; therefore, it is a patient safety issue. It is a near miss.
Found after treatment	Failure in the couch is an external fault that triggers an error in the patient. As soon as the error is observed, it is called a failure. This failure reached the patient, therefore, it is a patient safety issue, and failure is an incident, if it involves harm, it is an accident (adverse event).

Daily QA trending serves as a helpful example. For instance, the beam output deviates from the baseline every day. These daily deviations are observed but are not termed errors or failures, as we define an error as any deviation beyond the 5% threshold. If the deviation exceeds 5%, it is an error until a physicist examines the monitor and identifies the error beyond 5%. At that moment, it is externally observed and termed as a failure. If physicists do not look at the monitor, the error remains in the system as an error and continues to propagate until it ultimately manifests as a failure in patient outcome observations.

Within our community, we often refer to “preventing error”.^3^ As we discussed earlier, when we refer to an initial error, we mostly mean human error, where the root cause lies within the human factor, requiring the creation of conditions that minimize the likelihood of human error. What reports such as 394 refer to is what we call “preventing error propagation” here. Besides, “preventing error” carries a negative load and implies that errors are intrinsically evil. This is invalid in today's dynamic and adaptive systems. Errors are essential for a learning organization, as report 394 is indeed utilizing them for training and learning.^8^ Modern terms such as “managing errors” seem more suitable.

## AUTHOR CONTRIBUTIONS

The author was solely responsible for the conception, design, analysis, and writing of this manuscript.

## CONFLICT OF INTEREST STATEMENT

The author declares no conflicts of interest.

## ETHICS STATEMENT

This study did not involve human or animal subjects and, therefore did not require ethics approval.
